# 2949. Emergency Department (ED) Discharge Antibiotic Prescription Review by Hospital Pharmacists at a Large Academic Medical Center

**DOI:** 10.1093/ofid/ofad500.188

**Published:** 2023-11-27

**Authors:** Yanina Dubrovskaya, Cristian Merchan, Kassandra Marsh, Justin Siegfried, Vincent J Major, Jonathan So, James Rodriguez, Pradyuman Jhala, Eduardo Iturrate, John Papadopoulos, Dana Mazo, Samantha Smalley

**Affiliations:** NYU Langone Health, New York, New York; NYU Langone Health, New York, New York; NYU Langone Health, New York, New York; NYU Langone Health, New York, New York; NYULH, New York, New York; NYULH, New York, New York; NYU Langone Health, New York, New York; NYULH, New York, New York; NYU Langone Health, New York, New York; NYU Langone Health, New York, New York; New York University, New York University, NY; NYULH, New York, New York

## Abstract

**Background:**

Direct patient discharge from the Emergency Department (ED) presents an ideal time to promote antimicrobial stewardship (AS). On March 1, 2021 we initiated a pilot program where ED pharmacists conduct a review of discharge antibiotic prescriptions (abx Rxs). The aim of this program is to ensure appropriate discharge abxs. Willow Pharmacy Epic Analysts, ED and AS pharmacists collaborated on this program which included Epic integration of Rx Discharge Track Board, ED Discharge InBasket, ED Discharge Rx iVent and AS-led education sessions.

**Methods:**

Data for 2022 ED patients with discharge abx Rxs and iVents were extracted via Epic Reports. The first encounter per patient was included. We compared discharge abx Rxs and patient outcome data in Pharmacy vs No Pharmacy Review Groups (PRG, NPRG). Pharmacy iVents were used as a process measure.

**Results:**

Among 5747 ED adult patients with discharge abx Rxs, 2638 (46%) were reviewed by pharmacy (PRG). PRG and NPRG had similar prevalence of chronic pulmonary disease (12% vs. 13%), diabetes mellitus (9% vs. 10%), malignancy (8% vs. 9%), peripheral vascular disease (6% vs. 8%), renal disease (4% vs. 5%), and HIV (1% vs. 2%) despite a difference in mean Charlson Comorbidity Index (0.64 vs 0.75, p< 0.01). The primary ED diagnosis was skin and soft tissue, urinary, intra-abdominal infections and pneumonia (25 vs 23%, 18 vs 17%, 8 vs 9%, 5 vs 6%, respectively all p=NS). In PRG, discharge abxs demonstrated an increase use of penicillin (26 vs 24%, p=0.017) and 2^nd^ generation cephalosporin (17 vs 14%, p< 0.001). The PRG also decreased use of 3^rd^ generation cephalosporin (11 vs 12%, p=NS), quinolones (6 vs 7%, p=NS) and clindamycin (1.7 vs 2.4%, p=NS). There was no difference in ED length of stay (LOS 4.8h in each group), *C. difficile* infection (CDI) within 8 weeks (0.1 vs 0.2%, p=NS), and 30 days repeat ED visit or inpatient admission with the same diagnosis as index ED encounter (2.8 vs 2.6%, p=NS). Pharmacists intervened in 10% (272/2638) of ED abx Rxs with 92% (249/272) acceptance rate. The top 3 pharmacy interventions were recommending alternative abx (42%), dose optimization (31%) and abx duration (6%).

**Conclusion:**

Engaging pharmacists in ED discharge Rxs review resulted in use of narrower spectrum abx with lower CDI risk without any negative impact on ED LOS and clinical outcomes.

**Disclosures:**

**All Authors**: No reported disclosures

